# Establishment of Epstein–Barr Virus (EBV) Latent Gene-Expressing T-Cell Lines with an Expression Vector Harboring EBV Nuclear Antigen 1

**DOI:** 10.3390/microorganisms11112624

**Published:** 2023-10-25

**Authors:** Hiroyuki Kanno, Tomohiro Osada, Ayako Tateishi

**Affiliations:** Department of Pathology, Shinshu University School of Medicine, 3-1-1 Asahi, Matsumoto 390-8621, Japan; osdtmhr@gmail.com (T.O.);

**Keywords:** Epstein–Barr virus, chronic active EBV infection, EBV-encoded small RNAs, latent membrane protein 1, T-cell lines

## Abstract

Chronic active Epstein–Barr virus (EBV) infection (CAEBV) is characterized by chronic or recurrent infectious mononucleosis-like symptoms and is associated with EBV-associated T/natural killer (NK)-cell lymphoproliferative disorders, which frequently lead to the development of life-threatening complications, such as virus-associated hemophagocytic syndrome and EBV-positive apparent leukemia/lymphoma mainly in T- and NK-cell lineages. In order to clarify the EBV genes responsible for the diseases, we introduced the plasmid coding sequences of EBV-encoded small RNAs (EBERs) and/or latent membrane protein (LMP) 1 into human T-lymphocyte virus-I-negative human T-cell lines using a gene expression vector harboring EBV nuclear antigen 1, established the G418-resistant transformants of five T-cell lines, and quantitatively examined the expression of EBERs and LMP1 using real-time reverse transcriptase–polymerase chain reaction. The expression levels of EBERs in T-cell transformants with EBER DNA paralleled those in EBV-positive human T- and NK-cell lines, SNTK cells. The expression of LMP1 mRNA varied in SNTK cells and in human T-cell transformants, and the expression of LMP1 mRNA in T-cell lines expressing both EBERs and LMP1 was much lower than that in the same cell line expressing LMP1 mRNA alone. The currently employed gene expression system and currently obtained transformants may be useful for the analyses of the pathophysiology of CAEBV and EBV-positive T/NK-cell lymphoproliferative disorders.

## 1. Introduction

Epstein–Barr virus (EBV) is a ubiquitous herpesvirus in humans. Primary EBV infection is generally asymptomatic, but it sometimes causes infectious mononucleosis, which is basically self-limited. However, some individuals, mostly in East Asia, develop chronic infection with EBV. Chronic active EBV infection (CAEBV) is characterized by chronic or recurrent infectious mononucleosis-like symptoms and by high viral loads in peripheral blood [1,2], and it is associated with EBV-associated T/natural killer (NK)-cell lymphoproliferative disorders, including monoclonal proliferation [3], which frequently lead to the development of life-threatening complications, such as virus-associated hemophagocytic syndrome, probably due to the cytokinenemia caused by EBV-infected lymphoid cells [4], and cardiovascular diseases and vasculitis with the infiltration of EBV-infected lymphoid cells [1,5,6,7].

In CAEBV, the transcripts of EBV latent infection genes are detected in T or NK cells [2], and the in vitro EBV infection of human T-cell lines with the enforced expression of CD21, which is the EBV receptor in B-lineage cells, leads to the production of a macrophage-activating cytokine, tumor necrosis factor (TNF)α [8]. However, the EBV gene responsible for the expression of TNFα in T-lineage cells remains unclear. EBV infects a human T-lymphocyte virus-I (HTLV-I)-positive human T-cell line, MT-2 [9], and EBV-encoded small RNAs (EBERs) induce IL-9 in EBV-infected MT-2 cells [10]. However, infection with HTLV-I activates human T cells and induces the production of various cytokines [11]; thus, the change in cytokine production in HTLV-I-positive T-cell lines after superinfection with EBV might not reflect the function of EBV in vivo.

Profiles of the expression of the EBV latent infection genes in EBV-infected T and NK cells exhibit variations, and EBV nuclear antigen 1 (EBNA1), EBERs, *Bam*HI-A rightward transcripts (BARTs), latent membrane protein (LMP) 1, and LMP2s are expressed in infected T and NK cells in CAEBV [12,13]. EBNA1 enables efficient EBV episomal replication, transcription, and maintenance in latently infected dividing cells [14]; furthermore, EBNA1 has been shown to activate transcription from the episome, but not integrated DNA, and it might not affect host cellular gene expression [15]. 

In the current study, we aimed to establish cell models in which we could evaluate the role of EBV latent infection genes under a condition where the effects of other factors were minimized, which might affect host cellular gene expression. We thus introduced the plasmid coding sequences of EBERs and/or LMP1 into HTLV-I-negative human T-cell lines using a gene expression vector harboring EBNA1, and we established stable transformants.

## 2. Materials and Methods

### 2.1. Cell Lines and Culture

A lymphoblastoid cell line (LCL), TK6, was established via the infection of peripheral blood lymphocytes prepared from a healthy donor using Ficall-Paque^TM^ PLUS (GE Healthcare Bio-Sciences AB, Uppsala, Sweden) with EBV from B95-8 cells (a generous gift from Prof. K. Takada, Sapporo, Japan). HTLV-I-negative human T-cell lines, MOLT-14 and Peer, were gifts from Fujisaki Cell Center, Hayashibara Biochemical Labs., Inc. (Okayama, Japan). CCRF-HSB2, also an HTLV-I-negative human T-cell line, was a gift from the Health Science Research Resources Bank (Sennan, Japan). Human T-cell lines, Jurkat [16] and MOLT4 [17] (both HTLV-I-negative), were also used. These cells were cultured in RPMI 1640 medium supplemented with 10% heat-inactivated fetal bovine serum (MP Biomedicals, LLC, Solon, OH, USA). EBV-positive human T-cell lines, SNT8, SNT16, and SNT20, and EBV-positive NK-cell lines, SNK1, SNK6, and SNK10 (SNTK series: gifts from Dr. Norio Shimizu; Tokyo Medical and Dental University, Tokyo, Japan) [18,19,20], were grown in Artemis-2 medium (Nihon Techno Service, Ushiku, Japan). Geneticin (G418 sulfate) used for the selection of drug-resistant cells was purchased from Life Technologies (Grand Island, NY, USA). 

### 2.2. Plasmid Construction

A gene expression vector, pEBMulti-Neo, harboring *neo*R and EBNA1 derived from EBV, was purchased from Wako Pure Chemical Industries (Osaka, Japan). The 0.75 kb *Nhe*I-*Sac*I fragment of pEGFP-C1 containing the green fluorescence protein (GFP) open reading frame was cut out, and at the 5′-end of the fragment, an *Xho*I site was added; subsequently, the fragment containing the GFP open reading frame was subcloned into the *Xho*I site in multiple cloning sites (MCSs) of pEBMulti-Neo (pEBMulti-Neo-GFP). The 1.0 kb *Sac*I-*Eco*RI subfragment of the *Eco*RI K fragment (EKS) of Akata EBV DNA, which corresponds to the *Eco*RI J fragment of B95-8 EBV DNA and contains EBER1 and EBER2 reading frames [21], was cut out from pcDNA3-EK (a gift from Prof. K. Takada), which contained the *Eco*RI K fragment (EK) of Akata EBV DNA. At each end of the EKS fragment, a *Bgl*II or *Bam*HI site was added, and the *Bgl*II-*Bam*HI fragment containing the EKS fragment was concatenated six times in a direction-restricted fashion at the *Bam*HI site in the MCSs of pEBMulti-Neo (pEBMulti-Neo-EKS6) [21]. The 3.0 kb *Bam*HI fragment of pSV2gptMTLM (a gift from Prof. E. Kieff, Boston, MA, USA) containing EBV LMP1 cDNA [22] was cut out and subcloned into the *Bam*HI site in the MCSs of pEBMulti-Neo (pEBMulti-Neo-LMP1).

### 2.3. Transfection and Selection of Transfectants

For transfection, plasmid DNA was purified with a FlexiPrep kit (GE Healthcare, Uppsala, Sweden) or an illustra plasmidPrep Midi Flow kit (GE Healthcare, Buckinghamshire, UK). Plasmids were introduced to cell lines via electroporation. Then, 5 × 10^6^ cells were suspended in serum-free RPMI 1640 medium, washed twice, and resuspended in 600 μL of ice-cold medium containing 20 μg of plasmid DNA in a 4 mm gap electroporation cuvette. Electroporation was performed with Gene Pulser II (Bio-Rad, Hercules, CA, USA) at 1 mF and at the optimal voltage (210 V to 270 V) for each cell line [23]. The voltage of electroporation was optimized for each cell line by using pEBMulti-Neo-GFP and the percent of fluorescence-positive cells as an indicator. After electroporation, cells were cultured for 2 days, and transfectants were selected in the medium containing the appropriate concentration of Geneticin for each cell line. The optimal concentration of Geneticin for the selection of the transfectants of Jurkat, MOLT4, MOLT14, CCRF-HSB2, and Peer cells was 1000, 800, 800, 800, and 600 μg/mL, respectively. Subsequently, 2 × 10^4^ cells were seeded in a well in flat-bottomed 96-well culture plates with 200 μL of selection medium. Half of the medium was changed every 5 days until colonies emerged. Clones were expanded and maintained in the selection medium.

### 2.4. RNA Extraction and Real-Time Reverse Transcriptase–Polymerase Chain Reaction (RT-PCR)

Total RNA was extracted from the cell lines using TRIzol reagent (Life Technologies, Carlsbad, CA, USA), quantified by measuring OD_260_, and subsequently treated with DNase using a TURBO DNA-*free*^TM^ kit (Life Technologies, Carlsbad, CA, USA). For the reverse transcription of RNA samples, five μg of DNase-treated total RNA was converted to cDNA with a High-Capacity cDNA Reverse Transcription kit (Thermo Fisher Scientific, Vilnius, Lithuania) in a 50 μL reaction volume containing random hexamers according to the manufacturer’s protocol. For real-time PCR, a 0.5 μL aliquot of the cDNA sample (cDNA converted from 50 ng of total RNA) was diluted into 20 μL of a solution containing TaqMan^TM^ Fast Advanced master mix (Thermo Fisher Scientific, Vilnius, Lithuania) and each TaqMan assay reagent described below. Real-time PCR amplification was performed using the QuantStudio 3 real-time PCR system (Applied Biosystems, Foster City, CA, USA), according to the manufacturer’s protocol. 

For the absolute quantification of the copy numbers of EBERs, primers and TaqMan MGB probes were designed with Primer Express (v.1.5; Applied Biosystems) (Table 1). To check the specificity of each PCR amplification for EBER1 or EBER2, the PCR-amplified EBER1 and EBER2 sequences subcloned into the in vitro transcription vector for the preparation of an EBER1- or EBER2-specific RNA probe [24] were used as control DNA samples, and the specificity of each PCR amplification was confirmed; that is, the PCR for EBER1 did not amplify the EBER2 sequence, and the PCR for EBER2 did not amplify the EBER1 sequence (results not shown). Serial ten-fold dilutions of the plasmid DNA, pEBMulti-Neo-EKS6 with six copies of the EKS fragment of EBV DNA, which contained EBER1 and EBER2 reading frames, were used to obtain the standard curve of the absolute amount of each RNA, EBER1 or EBER2. The PCR of the cDNA samples was performed in triplicate, including serially diluted pEBMulti-Neo-EKS6 plasmid DNA as standard, and then the copy numbers of EBER1 and EBER2 in each cDNA sample were estimated. These PCR amplifications were repeated twice, and then we calculated the mean copy number of the total of six values in each sample.

For a relative quantification of LMP1 mRNA, the primers and TaqMan MGB probe were synthesized as previously described [25] (Table 1). A pre-developed TaqMan assay reagent for human glyceraldehyde 3-phosphate dehydrogenase (GAPDH) (TaqMan Gene Expression Assays; ID Hs99999905_m1) was used as the internal control for mRNA samples. Serial five-fold dilutions of the cDNA sample of TK6 were used to obtain the standard curve of the relative amounts of LMP1 mRNA. The PCR of the cDNA samples was performed in triplicate, including serially diluted cDNA samples of TK6 as standard, and then the relative amounts of LMP1 mRNA in each cDNA sample were estimated and represented as permillage of that in TK6 cells (LCLs). These PCR amplifications were repeated twice, and then we calculated the mean value of the total of six values in each sample.

## 3. Results

### 3.1. Establishment of Transformants

We could establish the G418-resistant transfectants of all five T-cell lines with the transfection of pEBMulti-Neo-GEP (Jurkat/G, MOLT4/G, MOLT14/G, CCRF-HSB2/G, and Peer/G), pEBMulti-Neo-EKS6 (Jurkat/E6, MOLT4/E6, MOLT14/E6, CCRF-HSB2/E6, and Peer/E6), pEBMulti-Neo-LMP1 (Jurkat/L, MOLT4/L, MOLT14/L, CCRF-HSB2/L, and Peer/L), or both pEBMulti-Neo-EKS6 and pEBMulti-Neo-LMP1 (Jurkat/E6 + L, MOLT4/E6 + L, MOLT14/E6 + L, CCRF-HSB2/E6 + L, and Peer/E6 + L). In the case of the transfection of both pEBMulti-Neo-EKS6 and pEBMulti-Neo-LMP1, 10 μg of each plasmid, totaling 20 μg, was used. For the analysis of RNA expression in transformants, cells were cultured for one week without G418; then, the cells were harvested, and the cell pellets were frozen quickly in liquid nitrogen and stored at −80 °C until use.

### 3.2. Expression of EBER1 and EBER2

**Figure 1:**
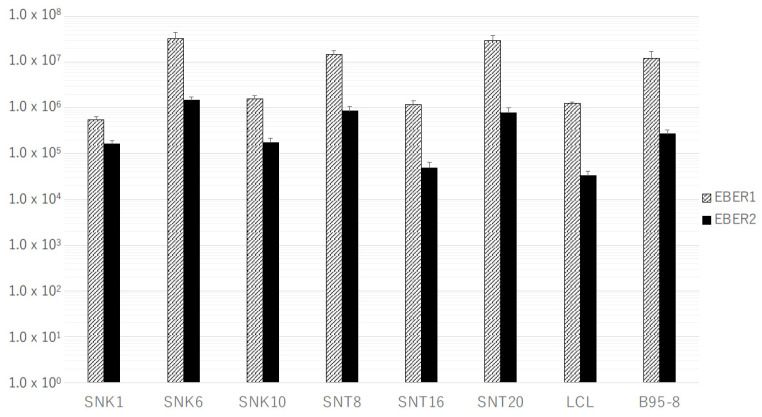
Expression of EBER1 and EBER2 in EBV-positive T- or NK-cell lines. Real-time RT-PCR specific for EBER1 or EBER2 was performed as described in Materials and Methods Section. Bars indicate the mean of absolute copy number of EBER1 or EBER2 in 50 ng of total RNA of each cell line.

**Figure 2:**
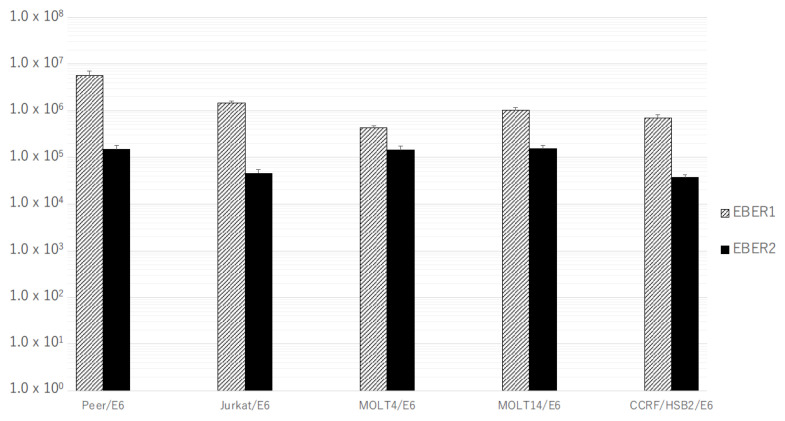
Expression of EBER1 and EBER2 in the stable transformants of human T-cell lines transfected with pEBMulti-Neo-EKS6. Real-time RT-PCR specific for EBER1 or EBER2 was performed as described in Materials and Methods Section. Bars indicate the mean of absolute copy number of EBER1 or EBER2 in 50 ng of total RNA of each transformant.

The expression levels of EBERs in SNTK cells showed variations, but, generally, they were equivalent to or much higher than those in LCLs. Furthermore, the copy number of EBER1 was much higher than that of EBER2 in all SNTK cell lines and LCLs (Figure 1). In all T-cell transformants transfected with pEBMulti-Neo-EKS6, both EBER1 and EBER2 were expressed at the same level or a little higher level than those in LCLs, and like SNTK cells and LCLs, the copy number of EBER1 was much higher than that of EBER2 (Figure 2). No expression of EBERs in mother T-cell lines without transfection was confirmed.

**Figure 3:**
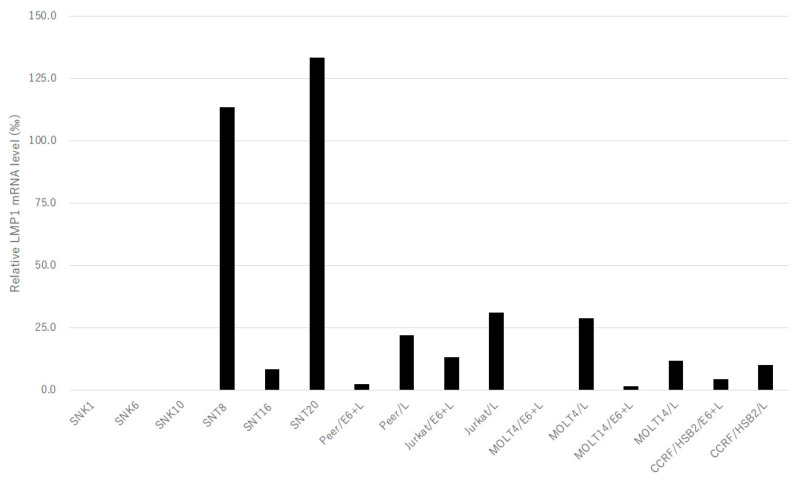
Expression of LMP1 mRNA in EBV-positive T- or NK-cell lines and in the stable transformants of human T-cell lines transfected with pEBMulti-Neo-LMP1 alone or both pEBMulti-Neo-EKS6 and pEBMulti-Neo-LMP1. Real-time RT-PCR specific for LMP1 mRNA was performed as described in Materials and Methods Section. Bars indicate the relative expression level of mRNA compared to that in TK6 cells, which was standardized with the expression of mRNA of glyceraldehyde-3-phosphate dehydrogenase, represented as permillage of that in TK6 cells (LCLs).

**Figure 4:**
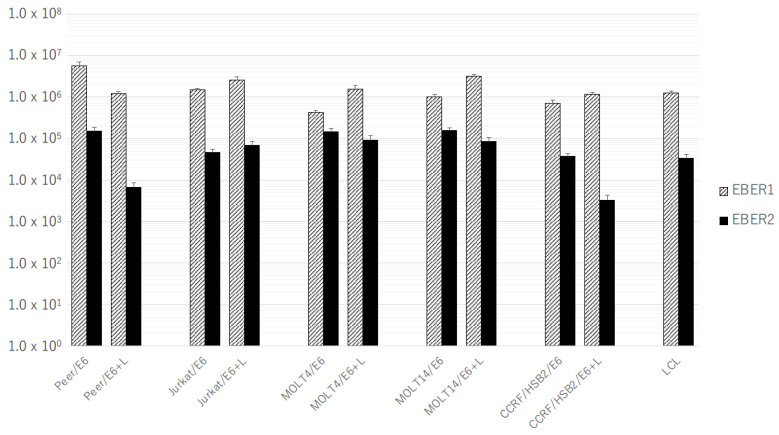
Expression of EBER1 and EBER2 in the stable transformants of human T-cell lines transfected with pEBMulti-Neo-EKS6 alone or both pEBMulti-Neo-EKS6 and pEBMulti-Neo-LMP1. Real-time RT-PCR specific for EBER1 or EBER2 was performed as described in Materials and Methods Section. Bars indicate the mean of absolute copy number of EBER1 or EBER2 in 50 ng of total RNA of each transformant.

Next, we examined the expression of EBERs in human T-cell lines with the co-expression of LMP1. The human T-cell transformants transfected with both pEBMulti-Neo-EKS6 and pEBMulti-Neo-LMP1 expressed LMP1 mRNA, even at a lower level than those with pEBMulti-Neo-LMP1 alone (Figure 3). The expression of EBER1 in T-cell lines generally increased with the co-expression of LMP1, except for in Peer cells. However, the expression of EBER2 in T-cell lines generally decreased with the co-expression of LMP1, except for in Jurkat cells (Figure 4).

### 3.3. Expression of LMP1 mRNA

The expression of LMP1 mRNA varied in SNTK cells and in human T-cell transformants, and it was not detected in SNK1, SNK6, or SNK10 cells (Figure 3). The expression levels of LMP1 mRNA in these cell lines were much lower than those in LCLs, less than one-seventh (Figure 3). No expression of LMP1 mRNA in mother T-cell lines without transfection was confirmed. Furthermore, the expression of LMP1 mRNA in T-cell lines expressing both EBERs and LMP1 (Jurkat/E6 + L, MOLT4/E6 + L, MOLT14/E6 + L, CCRF-HSB2/E6 + L, and Peer/E6 + L) was much lower than that in the same cell line expressing LMP1 mRNA alone (Jurkat/L, MOLT4/L, MOLT14/L, CCRF-HSB2/L, and Peer/L) (Figure 3). 

## 4. Discussion

In the current study, we used a pEBMulti-Neo vector to introduce EBV latent infection genes, established stable transformants of HTLV-I-negative T-cell lines expressing EBERs and/or LMP1, and further examined the amount of expressed RNAs using real-time RT-PCR. pEBMulti-Neo is a gene expression vector harboring EBNA1 derived from EBV; thus, it is distributed to daughter cells via the episomally replicating system. Therefore, we did not have to consider the effects of plasmid integration sites in chromosomal DNA on the expression of host genes, and, furthermore, this expression system may mimic EBV infection itself.

Both EBER1 and EBER2 were expressed in all the cell lines carrying EBER genes, EBV-positive T- or NK-cell lines, and human T-cell lines transfected with pEBMulti-Neo-EKS6, and the amount of expressed EBER1 was much higher than that of EBER2 in all the cell lines examined, including in LCLs and EBV-producing B95-8 cells (Figure 1 and Figure 2). The expression levels of EBERs in T-cell transformants with EBER DNA (Jurkat/E6, Jurkat/E6 + L, MOLT4/E6, MOLT4/E6 + L, MOTLT14/E6, MOLT14/E6 + L, CCRF-HSB2/E6, CCRF-HSB2/E6 + L, Peer/E6, and Peer/E6 + L) paralleled those in SNTK cells; thus, these transformants may be useful as model cell lines for the analyses of the function of EBERs in non-B-cell lineages. We had previously introduced a single copy of EBER genes into human T-cell lines in a site-directed manner with a Flip recombinase-mediated integration kit, Flip-In^TM^ system (Invitrogen, Carlsbad, CA, USA), and we obtained the transformants of MOLT14 cells expressing EBERs, but the amount of expressed EBER1 was much lower than that in an EBV-positive Burkitt’s lymphoma cell line, Akata [26]. Furthermore, we could not obtain the transformants of Jurkat or MOLT4 cells expressing EBERs [26]. Furthermore, differences in functions between EBER1 and EBER2 are reported in B-lineage cells [27]; thus, the real-time RT-PCR system established in this study may also be useful for further analyses of the exact functions of EBER1 and EBER2 in non-B-cell lineages.

Among the stable transformants established in this study, EBER1 expression generally increased with the expression of LMP1 mRNA, but EBER2 expression generally decreased with LMP1 mRNA (Figure 4). We did not examine the copy numbers of the expression plasmids introduced into T-cell lines; however, the copy numbers of the EBER1 gene and the EBER2 gene must be the same in a transformant. Thus, the effects of LMP1 on EBER1 expression may be different from those on EBER2. Further analyses in this regard might clarify the detailed mechanism of the expression of EBERs. 

The expression of LMP1 mRNA in T-cell transformants introduced with both of the expression vectors for LMP1 and EBERs was much lower than that in the same mother cell line introduced with the LMP1 gene alone (Figure 3). In a B-lineage cell line, BJAB, EBER2 interacts with host RNA-binding proteins that bind to B-cell transcription factor paired box protein 5 (PAX5) [28], and the knockdown of EBER2 results in the up-regulation of the expression of LMP2A/B and LMP1 [29]. Although we did not examine the copy number of the LMP1 expression vector in the transformants, this alteration in LMP1 expression was commonly observed in all five T-cell lines used in the current study. Therefore, we could point out the possibility that EBERs might also suppress the expression of LMP1 in T-lineage cells.

The currently established transformants have some limitations as a cell model for evaluating the role of EBV genes, since we cannot control the expression level of the introduced genes. The expression level of EBV genes varied among T-cell lines, as well as among SNTK cells, especially in LMP1 mRNA (Figure 1, Figure 2, Figure 3 and Figure 4). We are going to evaluate the functional alterations induced by EBV genes, such as resistance to apoptotic stimuli and cytokine production, including IL-9. In these analyses, however, the suppression of EBV RNA with siRNA or the drug-induced expression system must be employed in the evaluation. Furthermore, in addition to EBERs and LMP1, we will try to establish transformants expressing LMP2s and BARTs for a complete understanding of the regulation of T cells by EBV latent infection genes.

In the current study, we easily established stable transformants of human T-cell lines and observed the expression of EBV latent infection genes. The currently employed gene expression system and currently obtained transformants may be useful for the analyses of the pathophysiology of CAEBV and EBV-positive T/NK-cell lymphoproliferative disorders.

## Figures and Tables

**Table 1:** RT-PCR primers and probes for the quantification of EBV RNAs.

Primers and Probes	Sequences 5′-3′	Amplified Products
EBER1 forward	5′-GTGAGGACGGTGTCTGTGGTT-3′	58 bp
EBER1 reverse	5′-TTGACCGAAGACGGCAGAA-3′	
EBER1 probe	5′-TCTTCCCAGACTCTGC-3′	
EBER2 forward	5′-GCTACCGACCCGAGGTCAA-3′	77 bp
EBER2 reverse	5′-GAGAATCCTGACTTGCAAATGCT-3′	
EBER2 probe	5′-AAGAGAGGCTTCCCGCC-3′	
LMP1 forward	5′-CCACTTGGAGCCCTTTGTATACTC-3′	78 bp
LMP1 reverse	5′-TGCCTGTCCGTGCAAATTC-3′	
LMP1 probe	5′-ACTGATGATCACCCTCC-3′	

## Data Availability

Data sharing not applicable.
